# Concurrent Human Papillomavirus-Positive Squamous Cell Carcinoma of the Oropharynx in a Married Couple

**DOI:** 10.1155/2016/8481235

**Published:** 2016-06-22

**Authors:** Tyler D. Brobst, Joaquín J. García, Katharine A. Price, Ge Gao, David I. Smith, Daniel L. Price

**Affiliations:** ^1^Mayo Medical School, Mayo Clinic, Rochester, MN 55902, USA; ^2^Department of Anatomic Pathology, Mayo Clinic, Rochester, MN 55902, USA; ^3^Department of Medical Oncology, Mayo Clinic, Rochester, MN 55902, USA; ^4^Department of Laboratory Medicine and Pathology, Mayo Clinic, Rochester, MN 55902, USA; ^5^Department of Otolaryngology, Head and Neck Surgery, Mayo Clinic, Rochester, MN 55902, USA

## Abstract

*Background*. Although alcohol and tobacco use are known risk factors for development of squamous cell carcinoma in the head and neck, human papillomavirus (HPV) has been increasingly associated with this group of cancers. We describe the case of a married couple who presented with HPV-positive oropharynx squamous cell carcinoma within two months of each other.* Methods*. Tumor biopsies were positive for p16 and high-risk HPV in both patients. Sanger sequencing showed a nearly identical HPV16 strain in both patients. Both patients received chemoradiation, and one patient also underwent transoral robotic tongue base resection with bilateral neck dissection.* Results*. Both patients showed no evidence of recurrent disease on follow-up PET imaging.* Conclusions*. New head and neck symptoms should be promptly evaluated in the partner of a patient with known HPV-positive oropharynx cancer. This case expands the limited current literature on concurrent presentation of HPV-positive oropharynx squamous cell carcinoma in couples.

## 1. Introduction

Human papillomavirus (HPV), a sexually transmitted virus, is well known for its role in the development of cervical cancer in women. However, high-risk HPV—especially HPV16—has been increasingly associated with oropharynx squamous cell carcinoma (OPSCC) [1]. The overall increase in OPSCC in the United States from 1984 to 2004 has been attributed to increasing incidence of HPV-positive OPSCC. Indeed, the prevalence of HPV in oropharynx tumors increased from 16.3% in the 1980s to 72.7% in the 2000s [2], and oropharynx cancer is now the eighth most common cancer in men in the United States [3]. At the same time, the incidence of HPV-negative OPSCC has declined, reflecting a decline in smoking rates in the United States [2]. HPV-positive and HPV-negative tumors are associated with distinct sets of risk factors and have different tumor biology and natural history. While the most significant risk factor for HPV-negative OPSCC is alcohol and tobacco use [4], the risk of HPV-positive OPSCC is primarily related to sexual behavior, such as number of sexual partners and use of barrier methods. However, a history of smoking does have a negative impact on the prognosis of HPV-positive OPSCC [5]. HPV-positivity is a positive prognostic factor, as it is associated with reduced overall and disease-specific mortality compared to HPV-negative OPSCC [5, 6].

HPV appears to be more transmittable than other sexually transmitted viruses, and HPV infection is nearly ubiquitous among sexually active adults [7]. Although HPV infection among couples is common, it is rare for both partners to present simultaneously with HPV-positive OPSCC. Here we present the unusual clinical scenario of a married couple who presented with p16- and HPV-positive squamous cell carcinoma of the oropharynx within two months of each other.

## 2. Case Presentation

### 2.1. Patient A

60-year-old woman presented to otorhinolaryngology with one month of progressive otalgia, nasal congestion, odynophagia, and hemoptysis. Her medical history was significant for diabetes mellitus type II, hyperlipidemia, depression, gastroesophageal reflux disease, and obstructive sleep apnea. She had a tonsillectomy as a child and a significant smoking history of 25 pack-years. Examination revealed cervical lymphadenopathy and an ulcerated right tonsillar lesion extending across the palate and into the nasopharynx. A right tonsil biopsy revealed invasive squamous cell carcinoma that was positive for p16 by immunohistochemistry. In situ hybridization (ISH) showed the presence of diffuse high-risk HPV RNA. Positron emission tomography (PET) revealed prominent uptake in the right tonsil, with extension across the midline into the left tonsil and into the lateral nasopharynx, and involvement of the bilateral cervical lymph nodes without evidence of distant metastases (T4bN2cM0). Due to the extent of the primary tumor, she was not deemed a surgical candidate. She was treated with definitive concurrent chemoradiation therapy over 7 weeks in 35 fractions to 7,000 cGy of intensity modulated radiation therapy with weekly cisplatin. A PET scan performed 13 weeks after treatment showed no evidence of residual or recurrent squamous cell carcinoma, and physical examination corroborated a complete response. There was no evidence of recurrent disease at a 1-year follow-up visit.

### 2.2. Patient B

60-year-old man, Patient A's husband of 31 years, presented to family medicine with concern of a left supraclavicular mass that he had noticed two weeks before. His medical history was significant for hypertension, hyperlipidemia, hypothyroidism, coronary artery disease with drug-eluting stent placement, and chronic low back pain. He had a tonsillectomy as a child and had no smoking history but was exposed to second-hand smoke from his wife. Ultrasound imaging revealed the presence of multiple enlarged left supraclavicular lymph nodes. An excisional biopsy was performed, revealing metastatic squamous cell carcinoma that was positive for p16 by immunohistochemistry. In situ hybridization was positive for high-risk HPV RNA. This diagnosis was made approximately two months after his wife's diagnosis of HPV-positive squamous cell carcinoma. He was referred to otorhinolaryngology, where a smooth exophytic lesion was discovered at the left base of the tongue. Subsequent PET imaging suggested malignancy in the left base of tongue with metastases to the bilateral cervical lymph nodes without evidence of distant metastases (T2N2cM0). The patient underwent transoral robotic tongue base resection and bilateral neck dissections. Negative margins were achieved, and 9 of 76 lymph nodes were positive, the largest greater than 5 cm with gross extracapsular extension. He received a six-week course of adjuvant concurrent chemoradiation with 6,000 cGy in 30 fractions with weekly cisplatin. A PET scan performed 20 weeks after treatment showed no evidence of residual or recurrent squamous cell carcinoma. There was no evidence of recurrent disease at a 1-year follow-up visit.

### 2.3. p16 and HPV Detection

Surgical pathology archival material was histopathologically reviewed using hematoxylin and eosin-stained slides. Immunohistochemistry for p16INK4a was performed on formalin-fixed, paraffin-embedded tissue sections with the CINtec Histology Kit (Roche Diagnostics, Mannheim, Germany) using the automated Ventana BenchMark XT (Roche Diagnostics). RNA ISH for high-risk HPV E6/E7 mRNA was manually performed using the RNAscope HPV kit (Advanced Cell Diagnostics Inc., Hayward, California).

### 2.4. DNA Extraction, Polymerase Chain Reaction (PCR) Amplification, and Sanger Sequencing

DNA was extracted using the QIAamp DNA FFPE Tissue Kit (Qiagen, Hilden, Germany) following the manufacturer's protocol. End point PCR was performed using AmpliTaq Gold 360 master mix (Life Technologies, Carlsbad, California) and HPV16 L1 primers. The HPV16 L1 primer sequence is as follows: forward: 5′-CACAGTTATTCAGGATGGTGATATGG-3′, reverse: 5′-GAAGTAGATATGGCAGCACATAAT-3′. The PCR product was purified using the QIAquick PCR Purification kit prior to Sanger sequencing. Sanger sequencing was performed in both directions using both forward and reverse primers.

## 3. Discussion

**Figure 1:**
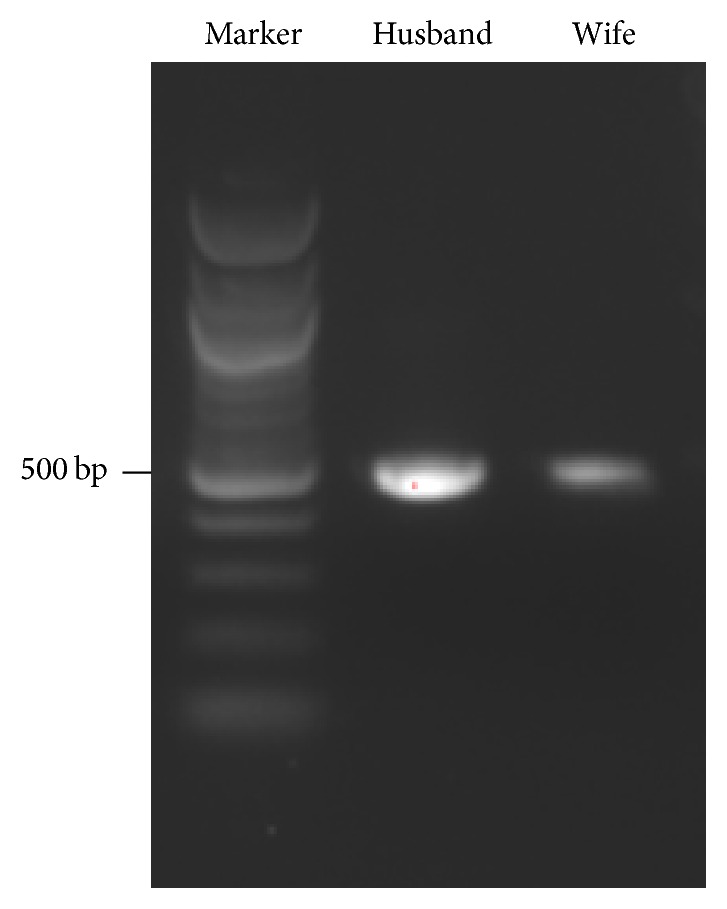
Southern blot of PCR product from HPV16 L1 region. A specific ~500 base pair HPV16 L1 PCR product was amplified from both husband and wife genomic tumor DNA.

This case presents the rare scenario of a married couple receiving a diagnosis of HPV-positive oropharynx squamous cell carcinoma within two months of each other. Given HPV's ubiquitous nature, it can often be difficult to say whether HPV was transmitted from one spouse to the other or whether each partner carried the infection before their relationship. In this case, genome analysis of tumor biopsies revealed that the couple was infected with a nearly identical strain of HPV16. We performed PCR amplification and Sanger sequencing to identify the presence of HPV sequence in both husband and wife. A specific ~500 bp HPV16 L1 PCR product was successfully amplified from both husband and wife genomic DNA (Figure 1). We then performed Sanger sequencing to identify the specificity of the virus; this showed that there was 99% identity between the husband virus sequence and the HPV16 virus sequence in the 450 bp region within L1 and there was 97% identity between the wife virus sequence and the HPV16 virus sequence in this 450 bp region. This demonstrates that both husband and wife were infected with an almost identical HPV16 strain, suggesting transmission of the virus between the couple.

HPV-induced genital warts exhibit a 60% risk of passing the infection to one's sexual partner, suggesting that HPV is highly transmittable. HPV appears to be more transmittable than other sexually transmitted viruses but about the same as sexually transmitted bacterial infections [7]. The synchronous presentation of HPV-positive OPSCC among couples has been reported in the past, but this appears to be an uncommon phenomenon given the relative paucity of literature published on the subject. In 2009, Andrews et al. reported two couples with HPV-positive tonsillar squamous cell carcinoma. In both couples, the partners presented with HPV-positive OPSCC within 12 months of each other [8]. A similar case of synchronous presentation of HPV-positive OPSCC in a couple was reported in 2008 by Haddad et al. [1]. Prior published cases of synchronous presentation of HPV-positive OPSCC among couples are summarized in Table 1 and compared with the patients presented here. D'Souza et al. examined HPV infection and cancer risk among partners of patients with known HPV-positive oropharynx cancer. They found that the presence of HPV16 DNA or HPV16 oncogene antibodies was rare among the partners, suggesting that their risk of HPV-positive oropharynx cancer was low [9].

The susceptibility of the oropharynx to HPV infection has not been fully explained. The invagination of the tonsillar mucosal surface may favor the retention of viral particles and their access to basal mucosal cells [10]. The method of carcinogenesis by HPV in the oropharynx is the same as that in the cervix; namely, the production of E6 and E7 proteins acts to inhibit the activity of the p53 and Rb tumor suppressors, respectively [1]. Overexpression of p16, a cyclin-dependent kinase inhibitor, is used to define a subset of HPV-positive tumors with favorable prognosis. Weinberger et al. found overall survival in patients with HPV/p16-positive tumors to be 79%, compared to 18% in patients with HPV-positive/p16-negative tumors [11]. Although the patients presented here had HPV- and p16-positive tumors, their long term outcomes may be negatively impacted by smoke exposure.

Although a rare occurrence, HPV-positive oropharynx squamous cell carcinoma can occur concurrently in couples. As shown in this case, the same HPV strain can be transmitted between a couple and lead to cancer in both partners. It is important to recognize that synchronous presentation of HPV-positive OPSCC in a couple does not necessarily imply that both partners carry the same viral strain or that one partner gave the infection to the other; indeed, it is possible that both partners carried the HPV infection before even meeting and then had similar time courses of cancer development. As demonstrated in this case, HPV genome amplification and DNA analysis may suggest transmission of the same viral strain between partners depending on genome similarity. HPV is a highly transmittable infection and is common among sexually active adults; the growing use of HPV vaccination will play an important role in combating HPV-positive oropharynx cancers. Prompt evaluation of new head and neck symptoms in the partner or spouse of a patient with a known HPV-positive oropharynx squamous cell carcinoma is warranted.

## Figures and Tables

**Table 1:** Published cases of synchronous presentation of human papillomavirus-positive oropharynx squamous cell carcinoma among couples.

		Haddad et al. [1]	Andrews et al. [8], Couple #1	Andrews et al. [8], Couple #2	This study
Partner 1	Sex	Female	Female	Female	Female
	Age	75	58	51	60
	Smoking history	Had 25 yr smoking history and quit 10 yrs ago	None	None	Current smoker of 25 yrs
	Alcohol use	None	None	None	Occasional
	Diagnosis	SCC	SCC	SCC	SCC
	Primary lesion	Right tonsil	Right tonsil	Left tonsil	Right tonsil
	TNM classification	T1 N1 M0	T1 N2b M0	T2 N1 M0	T4b N2c M0
	p16	p16+	p16+	p16+	p16+
	HPV	HPV16+	HPV16+	HPV16+	HPV16+
	Treatment	Not reported	Chemoradiation	Left neck dissection and postoperative radiation	Chemoradiation

Partner 2	Sex	Male	Male	Male	Male
	Age	75	56	57	60
	Smoking history	Had 12 yr smoking history and quit 25 yrs ago	Had minimal smoking history and quit 38 yrs ago	None	None
	Alcohol use	Drank heavily in past and quit 25 yrs ago	None	None	Occasional
	Diagnosis	SCC	SCC	SCC	SCC
	Primary lesion	Location unknown	Right tonsil	Right tonsil	Left base of tongue
	TNM classification	TX N2a M0	T1 N2a M0	T2 N2b M0	T2 N2c M0
	p16	Not reported	p16+	p16+	p16+
	HPV	HPV16+	HPV16+	HPV16+	HPV16+
	Treatment	Not reported	Chemoradiation and neck dissection	Chemoradiation	Transoral robotic tongue base resection, bilateral neck dissection, and chemoradiation

SCC: squamous cell carcinoma; HPV: human papillomavirus.
